# Supplementary material for: The causal relationship between the human gut microbiota and pyogenic arthritis: a Mendelian randomization study
Source: Front Cell Infect Microbiol. 2024 Nov 26;14:1452480. doi: 10.3389/fcimb.2024.1452480 (PMC11629706; doi:10.3389/fcimb.2024.1452480)
Supplement: Supplementary file 1 [file DataSheet1.zip › mendelian test/ebi-a-GCST90027785.csv_leave_one_out.pdf]

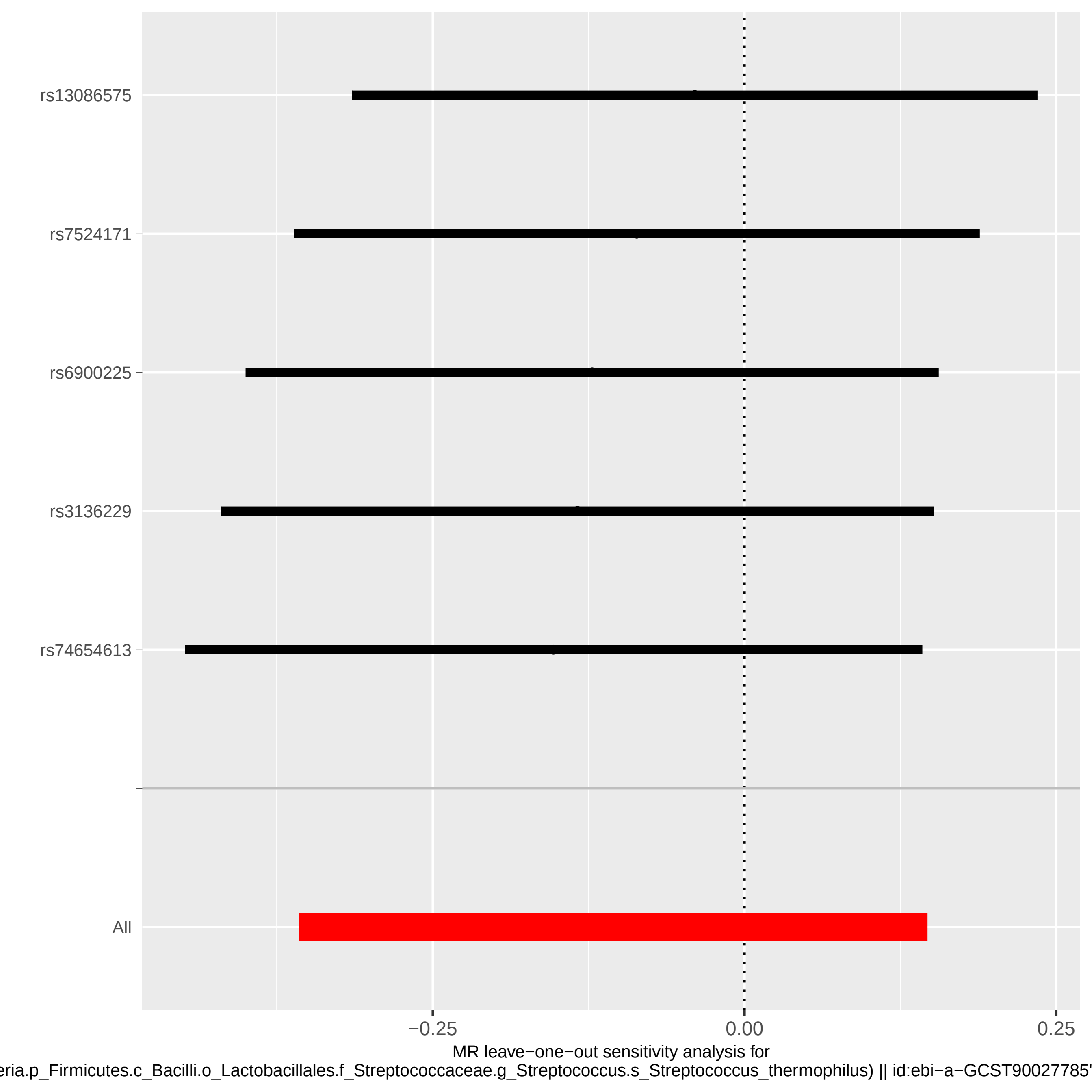
rs13086575
rs7524171
rs6900225
rs3136229
rs74654613
All
−0.25
0.00
0.25
MR leave−one−out sensitivity analysis for
eria.p_Firmicutes.c_Bacilli.o_Lactobacillales.f_Streptococcaceae.g_Streptococcus.s_Streptococcus_thermophilus) || id:ebi−a−GCST90027785
